# Outcomes and Trends of Prostate Biopsy for Prostate Cancer in Chinese Men from 2003 to 2011

**DOI:** 10.1371/journal.pone.0049914

**Published:** 2012-11-26

**Authors:** Rong Na, Haowen Jiang, Seong-Tae Kim, Yishuo Wu, Shijun Tong, Limin Zhang, Jianfeng Xu, Yinghao Sun, Qiang Ding

**Affiliations:** 1 Department of Urology, Huashan Hospital, Fudan University, Shanghai, China; 2 Center for Cancer Genomics, Wake Forest University School of Medicine, Winston-Salem, North Carolina, United States of America; 3 Fudan Institute of Urology, Huashan Hospital, Fudan University, Shanghai, China; 4 Department of Urology, Changhai Hospital, Shanghai, China; Innsbruck Medical University, Austria

## Abstract

**Background:**

Prostate-specific antigen (PSA) screening is growing in popularity in China, but its impact on biopsy characteristics and outcomes are poorly understood.

**Objective:**

Our objective was to characterize prostate biopsy outcomes and trends in Chinese men over a 10-year period, since the increasing use of PSA tests.

**Methods:**

All men (n = 1,650) who underwent prostate biopsy for PCa at Huashan Hospital, Shanghai, China from 2003–2011 were evaluated. Demographic and clinical information was collected for each patient, including age, digital rectal examination (DRE), transrectal ultrasound (prostate volume and nodule), total prostate-specific antigen (tPSA) levels and free PSA ratio (fPSA/tPSA) prior to biopsy. Prostate biopsy was performed using six cores before October 2007 or ten cores thereafter. Logistic regression and multivariate analysis were used to evaluate our data.

**Results:**

The overall positive rate of prostate biopsy for PCa was 47% and the rate decreased significantly over the years from 74% in 2003 to 33% in 2011 (P-trend = 0.004) . Age at diagnosis was slightly increased (P-trend = 0.04) while fPSA/tPSA was significantly decreased (P-trend = 1.11×10-5). A statistically significant trend was not observed for tPSA levels, prostate volume, or proportion of positive nodule. The model including multiple demographic and clinical variables (i.e., age, DRE, tPSA, fPSA/tPSA and transrectal ultrasound results) (AUC = 0.93) statistically outperformed models that included only PSA (AUC = 0.85) or fPSA/tPSA (AUC = 0.66) to predict PCa risks (P<0.05). Similar results were observed in a subgroup of men whose tPSA levels were lower than 20 ng/mL (AUC = 0.87, vs. AUC of tPSA  = 0.62, P<0.05).

**Conclusions:**

Detection rates of PCa and high-grade PCa among men that underwent prostate biopsy at the institution has decreased significantly in the past 10 years, likely due to increasing use of PSA tests. Predictive performance of demographic and clinical variables of PCa was excellent. These variables should be used in clinics to determine the need for prostate biopsy.

## Introduction

In the past two decades, prostate cancer (PCa) has become one of the most prevalent malignant tumors in western countries, and is the second leading cause of cancer death in men [1]. In China, the incidence of PCa has risen significantly in recent years, however, prostate-specific antigen (PSA) screening is not common and the majority of Chinese patients are found to have high grade PCa at diagnosis. Before 2007, standard guidelines for PSA testing did not exist in China. PSA testing was provided based on clinician's experience. For example, PSA testing prior to 2007 was only offered to Chinese men highly suspected of having PCa. Specifically, clinician's recommended testing if a patient had urinary tract symptoms, a positive digital rectal exam (DRE) or positive transrectal ultrasound. While urology guidelines in China after 2007 recommended annual PSA testing for men over 55 years, testing was selectively provided, most commonly due to lack of insurance coverage. Because PSA testing is not routine in China, limited data was available to set tPSA thresholds for prostate biopsy. In this study, our objective was to characterize prostate biopsy outcomes and trends in Chinese men over a 10-year period, since the introduction of PSA tests in China.

## Materials and Methods

### 2.1 Patient population

Our study included all patients (n = 1,650) who underwent prostate biopsy for PCa during 2003 to 2011 at Huashan Hospital, Fudan University in Shanghai, China (Table 1). As a tertiary health institute, Huashan Hospital provides a highly technical level of medical health care and research, especially for cancer, special clinical procedures, and other uncommon and severe diseases. Although most tertiary health institutes like Huashan Hospital are located in metropolitan areas of China, patients from all over the country seek their services. Before October 2007, standard indications were not available for prostate biopsy in China. However, after that, the indications for prostate biopsy in our institution were: (1) tPSA>4.0 ng/mL (not the first elevated PSA, but the PSA after weeks of surveillance and confirmation; in addition, the patient should meet standard criteria, i.e. no ejaculation and no manipulations such as catheterization, cystoscopy or transurethral resection, and no urinary tract infections) (2) tPSA<4.0 ng/mL, with suspicious fPSA/tPSA (<0.16) or PSAD (>0.15); (3) Positive findings from DRE, with any level of tPSA; (4) Positive findings from imaging techniques, such as transrectal ultrasound and magnetic resonance imaging (MRI), with any level of tPSA.

**Table 1:** Characteristic table of prostate biopsies performed from 2003–2011 in Huashan Hospital, Shanghai, China.

	2003	2004	2005	2006	2007	2008	2009	2010	2011	P-trend	All cases
No. of biopsies	58	85	268	294	180	186	196	175	208	/	1650
PCa (%)	74.	60	50	43	46	39	42	50	33	0.004	47
Gleason score≥8 (%)	—*	50	61	51	46	42	32	40	27	2.08E-07	44
Age of entire group (SD) (Yr)	72.02 (7.22)	71.09 (8.76)	70.07 (8.02)	71.25 (7.99)	71.81 (9.15)	71.65 (8.48)	71.98 (9.15)	70.27 (9.17)	71.66 (9.32)	/	71.24 (8.65)
Age of PCa group (SD) (Yr)	71.65 (7.42)	72.49 (8.25)	71.13 (7.69)	72.95 (7.84)	72.41 (9.2)	72.82 (8.86)	75.69 (7.93)	72.89 (8.64)	74.72 (8.24)	0.040	72.96 (8.30)
Age of non-PCa group (SD) (Yr)	73.07 (7.76)	69.00 (9.21)	68.99 (8.22)	69.99 (7.89)	71.31 (9.13)	70.88 (8.17)	69.35 (9.08)	67.68 (8.98)	69.13 (9.42)	/	69.76 (8.69)
tPSA° (SD) (ng/mL)	52.67 (3.44)	62.04 (3.07)	48.96 (2.42)	51.76 (2.33)	76.81 (5.22)	47.86 (3.95)	56.5 (4.24)	56.67 (4.13)	42.72 (4.23)	0.470	53.55 (3.54)
fPSA/tPSA (SD)	0.15 (0.17)	0.19 (0.21)	0.2 (0.16)	0.2 (0.16)	0.12 (0.08)	0.12 (0.08)	0.12 (0.07)	0.13 (0.07)	0.12 (0.09)	1.11E-05	0.15 (0.13)
Prostate Volume° (SD) (mL)	39.37 (1.57)	42.75 (1.62)	43.65 (1.66)	43.35 (1.64)	46.08 (1.63)	41.91 (1.53)	41.27 (1.6)	45.54 (1.64)	41.21 (1.61)	0.301	43.05 (1.62)
Nodule (%)	85.29	76.67	80.18	81.9	86.76	81.82	84.21	80.72	75.58	0.507	81.34

* No Data about Gleason Scores was available in 2003.

### 2.2 Sample collection

All patients underwent transrectal ultrasound guided transperineal prostate biopsy and had 6 core biopsies before Oct. 2007 or 10 core biopsies after Oct. 2007. All specimens were diagnosed by doctors in the Pathology Department of Huashan Hospital. Blood samples were collected on the day before biopsy and prior to any manipulations (e.g. DRE, transrectal ultrasound) that may have caused a transient increase of biomarkers. The samples were temporarily stored in a serum tube and sent immediately to the Department of Clinical Laboratory. We used the same method to measure tPSA and fPSA. Written informed consent was obtained from each patient for their participation and so that their information could be stored in the hospital database and used for research. The study was approved by the Institutional Review Board of Huashan Hospital, Fudan University, Shanghai China.

### 2.3 Statistic analysis

Univariate and multivariate logistic regression models were used to predict PCa and high grade PCa. Demographic and clinical variables used in the models included age, logarithm of tPSA, fPSA/tPSA, logarithm of prostate volume, result of DRE, and result of transrectal ultrasound. The predictive performance for each model (different combinations of the variables above) was measured using the area under the receiver operating curve (AUC). Statistical analyses were implemented using PROC LOGISTIC in SAS 9.2 (SAS Institute, Cary, NC).

## Results

A total of 774 out of the 1,650 (47%) patients that underwent biopsies were diagnosed with PCa (PCa group). The total positive rate of biopsy was 47% and significantly decreased over the study period (*P_trend_* = 0.004, Table 1). The mean age of all men was 71.24 years. Patients with PCa (72.96 years) were older than patients with other diseases (non-PCa group) (69.76 years, p<0.001). Age at diagnosis was increased slightly from 71.65 years in 2003 to 74.72 years in 2011 (*P_trend_* = 0.04, Table 1).

**Figure 1:**
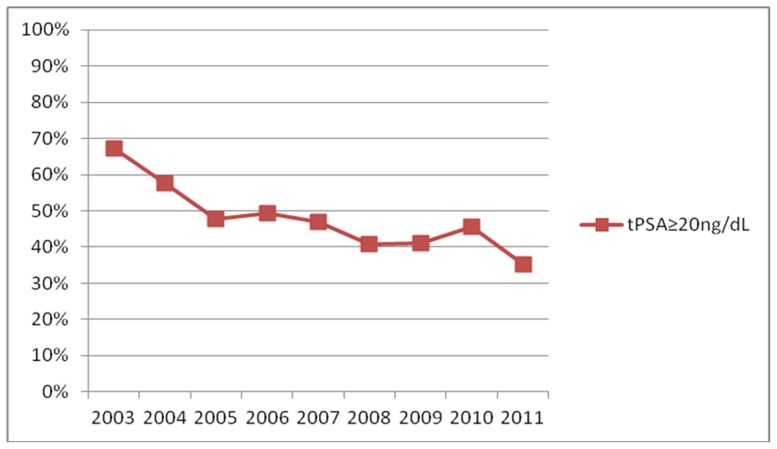
The percentage of tPSA levels over 20 ng/mL in PCa patients. There was a significant decrease in the percentage of tPSA levels over 20 ng/mL in PCa patients from 2003 to 2010 (*P_trend_* = 2.26×10^−5^).

Compared to the non-PCa group, the PCa group had much higher tPSA levels (mean: 53.61 ng/mL vs. 11.9 ng/mL, p<0.001) and lower fPSA/tPSA levels (mean: 0.15 vs. 0.19, p<0.001). There was a significant decrease in fPSA/tPSA levels (*P_trend_* = 1.11×10^−5^, Table 1) and the percentage of tPSA levels for PCa patients with levels over 20 ng/mL (*P_trend_* = 2.26×10^−5^, Fig. 1), while no statistically significant change in trend for patients with tPSA levels under 20 ng/mL (*P_trend_* = 0.47, Table 1) was observed. The overall positive prostate biopsy rates were 14.8% for tPSA<10 ng/mL, 27.4% for tPSA≥10 ng/mL and <20 ng/mL, and 75.8% for tPSA≥20 ng/mL. We observed a similar trend for high-grade PCa (Gleason Score≥8).

**Figure 2:**
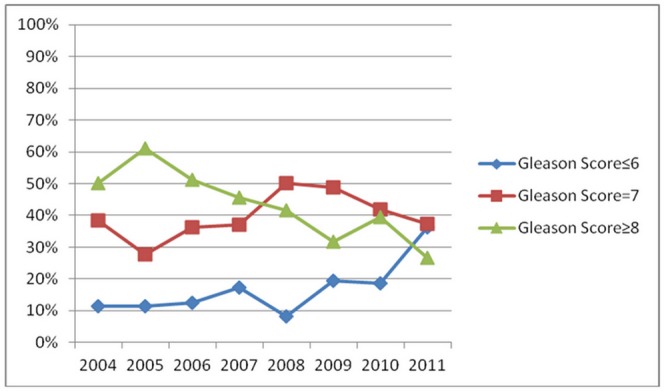
Trends of Gleason Score ≤6,  = 7, ≥8 Groups over the years. The percentage of ≥8 group trend to go downwards from 50% in 2004 to 27% in 2011 (*P_trend_* = 2.08×10^−7^).

Among 774 PCa patients, 670 patients had complete Gleason score information. Most of them had biopsies from 2004 to 2011 (Only 3 out of 58 men (43 PCa) in 2003 had the information of Gleason Score). The majority of patients had Gleason scores ≥8 (Mean: 43.58% for Gleason Score≥8, 38.81% for Gleason Score = 7, 17.61% for Gleason Score≤6), the percentage of patients with Gleason scores≥8 decreased from 50% in 2004 to 27% in 2011 (*P_trend_* = 2.08×10^−7^, Table 1, Fig. 2). This suggests that men have been getting diagnosed with PCa at earlier stages during the past ten years.

We first performed univariate analysis to test the association between PCa and each variable (age, tPSA, fPSA/tPSA, volume, nodule, DRE) (Table 2). A comparison of prostate volumes by transrectal ultrasound revealed that the PCa group (Mean = 43.06 mL) had significantly (p<0.001) smaller prostate volumes than those of the non-PCa group (Mean = 54.3 mL). In addition, the PCa group (Mean = 81.24%) was twice as likely (p<0.001) to have one or more nodules than the non-PCa group (Mean = 40.58%). Also, the PCa group had a 5-fold higher percentage for abnormal DRE (PCa 56.85% vs. non-PCa 10.43%, p<0.001). The AUCs ranged from 0.610 for the model with age only to 0.847 for the model with tPSA only.

**Table 2:** Univariate analysis (testing the association between each variable and PCa).

	Age(Mean(SD))	tPSA°(ng/mL, Mean(SD))	fPSA/tPSA° (Mean(SD))	Volume(mL)	Nodule(%)	DRE(%)
PCa	72.94(8.30)	53.61(3.55)	0.15(0.13)	43.06(1.62)	81.24	56.85
non-PCa	69.76(8.69)	11.9(2.22)	0.19(0.12)	54.3(1.57)	40.58	10.43
P-value (PCa vs. non-PCa)	2.02E-13	6.05E-85	2.56E-06	2.81E-20	1.69E-51	2.65E-81
OR(95%CI)	1.05 (1.03–1.06)	4.81 (4.11–5.63)	0.08 (0.03–0.23)	0.34 (0.27–0.43)	6.34 (4.99–8.06)	12.9 (9.89–16.7)
AUC	0.610	0.847	0.663	0.645	0.703	0.751

° The tPSA and fPSA values are antilogarithmic when calculating P-value, OR and AUC.

Next, we stratified our data into three groups, a group with all men (entire group), a group of men with tPSA<10 ng/mL (tPSA<10 ng/mL group) and a group of men with tPSA<20 ng/mL (tPSA<20 ng/mL group), calculating AUC by using tPSA only (as shown before, tPSA itself performed well in predicting PCa) and two types of multivariate models (model 1: modeling by using 5 variables of age, logarithm of tPSA, logarithm of prostate volume, result of DRE, result of transrectal ultrasound; model 2: added fPSA/tPSA besides the five variables). The multivariate models increased the prediction value for both PCa and high grade PCa. In the tPSA<10 ng/mL group, multivariate models didn't perform better comparing with tPSA only, probably because the number of the high-grade PCa samples was too small. By using tPSA only, we got significant different AUCs, with 0.85 in the entire group, 0.62 (P<0.05) in the tPSA<20 ng/mL group and 0.57 (P<0.05) in the tPSA<10 ng/mL group, which showed a decrease of AUCs when we adjusted the tPSA threshold to a lower level. We observed similar results by using multivariate models, with 0.93, 0.86 (P<0.05) and 0.87 (P<0.05) by model 1, and with 0.93, 0.87 (P<0.05) and 0.87 (P<0.05) by model 2 in the entire, tPSA<20 ng/mL and tPSA<10 ng/mL groups respectively. However, multivariate models predicted almost equally for PCa in the tPSA<20 ng/mL and tPSA<10 ng/mL groups when using these models (Table 3). Generally, multivariate models had better prediction utility than tPSA only for PCa (P<0.05). When predicting high-grade PCa (Gleason Score≥8), multivariate models outperformed the model that only used tPSA in the entire group and tPSA<20 ng/mL group (P<0.05), but performed equally in the tPSA<10 ng/mL group (P>0.05) (Table 3).

**Table 3:** Performance of tPSA only and Multivariate model for predicting PCa and high grade PCa in different stratification.

Group	Model	PCa	High Grade PCa (Gleason Score≥8)
		AUC (Area Under the Curve)	
Total men (PCa: 774 vs. non-PCa: 876) (High grade PCa: 290)	tPSA only	0.85	0.62
	*Multivariate Model 1	0.93	0.66
	**Multivariate Model 2	0.93	0.67
Men with tPSA<20 ng/mL (PCa:198 vs. non-PCa: 692) (High grade PCa: 58)	tPSA only	0.62	0.54
	Multivariate Model 1	0.86	0.63
	Multivariate Model 2	0.87	0.63
Men with tPSA<10 ng/mL (PCa:59 vs. non-PCa:325) (High grade PCa: 18)	tPSA only	0.57	0.92
	Multivariate Model 1	0.87	0.87
	Multivariate Model 2	0.87	0.94

* Multivariate Model 1: Modeling by using 5 variables: age, logarithm of tPSA, logarithm of prostate volume, result of DRE, result of transrectal ultrasound.

** Multivariate Model 2: Modeling by using 6 variables: age, logarithm of tPSA, fPSA/tPSA, logarithm of prostate volume, result of DRE, result of transrectal ultrasound.

-The tPSA and fPSA values are antilogarithmic when calculating AUC.

**Figure 3:**
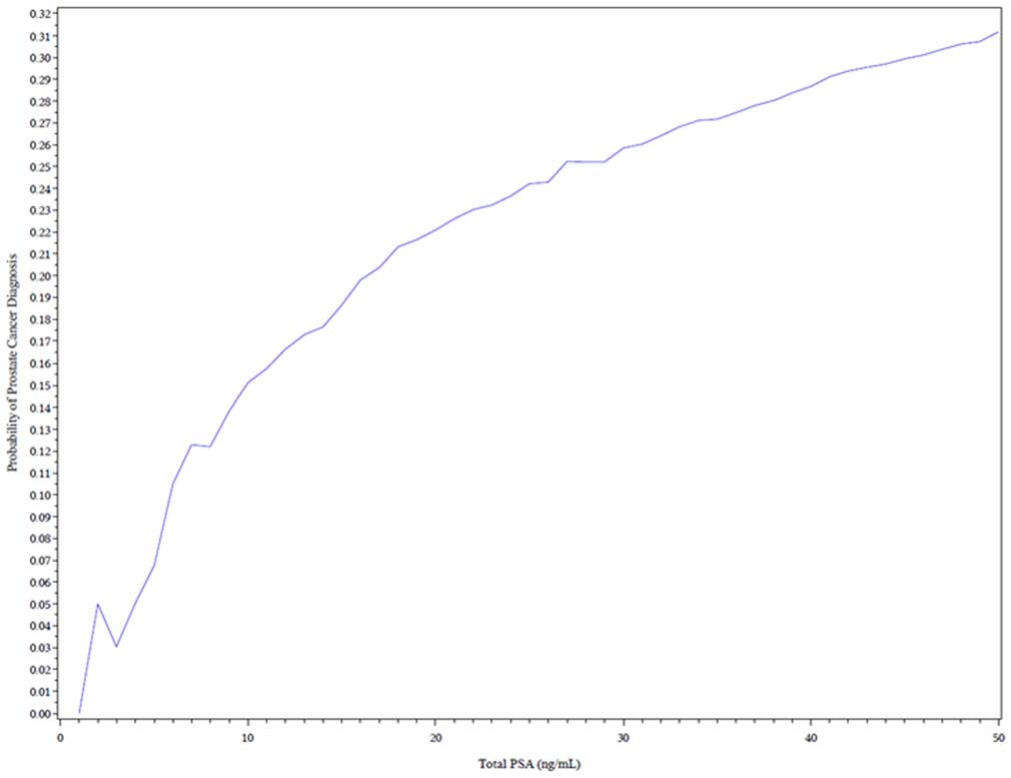
Probability of having prostate cancer with increasing PSA in biopsy population. The probability of having prostate cancer when undertaking prostate biopsy is raised from nearly 0% with tPSA<1.0 ng/mL to nearly 31% with tPSA = 50 ng/mL according to our study population. This figure can be use to predict the probability with tPSA level in the future.

The total risk of PCa at our institute was 47% (positive rate of prostate biopsy). We calculated the risk at different PSA levels. The risk of PCa ranged from 4.7% to 14.8% with the tPSA level from 4 ng/mL to 10 ng/mL. Men with tPSA levels of 20 ng/mL had a risk of 22.4% (Fig. 3). We also evaluated the sensitivity and specificity at different cutoff PSA levels (Table 4.). We found that when the cutoff value was 4 ng/mL, the sensitivity was 99.7%, and the specificity was 4.4%. When the cutoff value was 10 ng/mL, the sensitivity would be 92.4%, while the specificity rose to 37.3%. If we increase the cutoff value to 20 ng/mL, the sensitivity decreases to 74.3%, however, the specificity raises to 79.4%.

**Table 4:** Different sensitivities and specificities in different tPSA cutoff level.

		Sensitivity (%)	Specificity (%)
Cutoff tPSA value (ng/mL)	4	99.7	4.4
	10	92.4	37.3
	12	88.4	52.9
	15	82.7	67.5
	20	74.3	79.4

## Discussion

To the best of our knowledge, this is the first retrospective study to evaluate the prevalence and trend of biopsy use for PCa after the increasing use of PSA screening in a Chinese population. We also evaluated the predictive performance of variables for PCa and high-grade PCa.

Most studies conducted in western countries had positive detection rates <35%, much lower than the rates (47%) we observed in our study. However, some of the previous studies had large populations and were based on randomized screening trials [2]–[5]. In addition, most of those studies used tPSA levels of 1.25 ng/mL–2.5 ng/mL as their cutoff values to perform prostate biopsy, compared with the cutoff value of tPSA>4 ng/mL used in our study. Therefore, the detection rates reported in those studies are not comparable to our study. One the other hand, there are a few studies that used single institute cohorts including New York Presbyterian Hospital (Weill Medical College of Cornell University, New York, NY), Cleveland Clinic (Cleveland Clinic, Cleveland, OH, USA) and Durham VA (Durham VA Medical Center, Durham, NC, USA). The detection rates from those studies were 31%, 39% and 47% respectively, which were more comparable to ours [4]–[7]. The positive rate of prostate biopsy has decreased over the years at our institute. Zhu et al. (2009) observed similar findings in a Chinese population [8]. This may be due to the fact that PSA testing was not popular in China during earlier years and patients were more likely to be biopsied because they were experiencing other symptoms (e.g. hematuria, dysuria). Therefore, they observed higher detection rates of PCa and dropped thereafter due to widespread use of PSA test.

According to results from studies conducted in western countries, the risk for developing PCa varies from 15% (Goteborg cohort) to 40% (SABOR cohort) when a tPSA threshold of 4 ng/mL is used [2]. Our study showed that only 4.7% of men with a tPSA level of 4 ng/mL were diagnosed with PCa, much lower than rates in western countries. Even men with a tPSA level of 10 ng/mL had lower risk (14.8%) than men that participated in western studies. In addition, according to our data, although the sensitivity for tPSA = 10 ng/mL was 92.4%, lower (P<0.05) than that for 4 ng/mL (99.7%), the specificity for tPSA = 10 ng/mL (37.3%) was much higher (P<0.05) than that for tPSA = 4 ng/mL (4.4%). Using a cutoff value of 4 ng/mL for prostate biopsy will cause a large number of men to undergo unnecessary prostate biopsies. Thus, we believe that using a cutoff value of tPSA>4 ng/mL for prostate biopsy in China is not appropriate. We suggest that when using tPSA>4 ng/mL as cutoff value for prostate biopsy, fPSA/tPSA, PSAD or other clinical information should be comprehensively considered before a new cutoff value is set up based on further prospective and larger population studies.

The tPSA levels at diagnosis for Chinese men were much higher than levels for men in western countries. Our tPSA level at diagnosis fluctuated from 28.6 ng/mL to 50.9 ng/mL (median), while in western trials the median ranged from 11.8 ng/mL to 6.3 ng/L [9]. We also found that the percentage of patients with Gleason scores >8 was 43.58%, much higher than those of studies from western countries, which ranged from 2% (Goteborg cohort) to 21% (Tyrol cohort ) [2]. Overall, the downward trend of high-grade PCa percentages shows that there are benefits to PSA screening.

The SEER database documented a decline in age at diagnosis from 72 to 69.4 yr from 1990 to 1994 due to the increasing use of PSA testing and increased ability of early detection of serum PSA [10]. However, our data showed a different result with a slight increase of age at diagnosis from 2003 to 2011 (Table 1). This may be due to the fact that, with the increasing use of PSA testing, clinicians were more likely to monitor or use active surveillance of PSA levels instead of performing prostate biopsies with initial abnormal PSA results.

Studies in the tPSA era have demonstrated a fall in PSA levels at diagnosis [11]. The median tPSA level at the time of diagnosis decreased from 11.8 ng/mL in 1990 to 6.3 ng/mL in 1998 [10]. The levels of tPSA over the years didn't show a significant trend, however, because of the significant decrease of percentage of patients with tPSA>20 ng/mL, we can still conclude that PSA screening had benefits over the years.

We would like to point out that there were two notable changes in the clinical parameters during the study period. First, we obtained 6 cores for prostate biopsy before Oct. 2007 and 10 cores thereafter. Up to a 40% increase in detection rates of PCa were reported in some of the studies when they took extra biopsy cores [12]–[18]. However, other studies didn't reach this same conclusion [19]–[22]. In our study, we did not observe a significant difference between detection rates based on 6-core biopsy and 10-core biopsy (Chi-square test, P = 0.976). Thus, the number of cores was not included in our multivariate analysis. Second, the International Society of Urological Pathology modified the Gleason scoring system in 2005, thereby introducing some potential bias [23]–[27]. However, this had limited effects on our study as only 8.7% of our study population were graded using the old Gleason score system before 2005. More importantly, our analysis of high-grade PCa was defined as Gleason score ≥8, and men in this category were least affected by the new scoring system [23]–[27].

Although retrospective, our study presents a good depiction of PCa prevalence and the trends of prostate biopsy in Chinese men. Some of the limitations of this study include the lack of family history and that study participants were recruited from a single institution. Although most tertiary health institutes like Huashan Hospital are located in metropolitan areas of China, patients from all over the country seek their services.

## Conclusions

Detection rates of PCa and high-grade PCa among men that underwent prostate biopsy in China have decreased significantly in the last 10 years. This trend is likely due to the increasing use of PSA testing. Significant differences in positive prostate biopsy rates were found between western countries and China. Predictive performance of demographic and clinical variables of PCa was excellent. These variables should be used in clinics to determine the need for prostate biopsy. Furthermore, the cutoff value of 4 ng/mL for prostate biopsy in China is not appropriate, and should be considered in further studies.
